# Neutralizing antibodies against SARS-CoV-2 in Brazilian pregnant women vaccinated with one or two doses of BNT162b2 mRNA vaccine (Pfizer/Wyeth^TM^)

**DOI:** 10.3389/fpubh.2022.1054460

**Published:** 2023-01-04

**Authors:** Mauro César da Silva, Neila Caroline Henrique da Silva, Ana Laura Carneiro Gomes Ferreira, Fernanda Carneiro Gomes Ferreira, Maria Inês Bezerra de Melo, Letícia Micherlyne Xavier da Silva, Camila Rodrigues de Melo Barbosa, Jurandy Júnior Ferraz de Magalhães, George Tadeu Nunes Diniz, Ariani Impieri Souza, Norma Lucena-Silva

**Affiliations:** ^1^Laboratory of Immunogenetics, Department of Immunology, Aggeu Magalhães Institute, Oswaldo Cruz Foundation, Recife, Brazil; ^2^Women Health Research Group of Instituto de Medicina Integral Prof. Fernando Figueira, Recife, Brazil; ^3^University of Pernambuco, Recife, Brazil; ^4^Pernambuco College of Health (FPS), Instituto de Medicina Integral Prof. Fernando Figueira (IMIP), Recife, Brazil; ^5^Clinical Hospital, Federal University of Pernambuco, Recife, Brazil; ^6^Central Laboratory of Pernambuco, State Secretary of Health, Recife, Brazil; ^7^Laboratory of Computational Methods, Aggeu Magalhães Institute, Recife, Brazil

**Keywords:** COVID-19, pregnancy, vaccines, neutralizing antibodies, IgG, SARS-CoV-2

## Abstract

Pregnant women have an increased risk of developing severe coronavirus disease. In Brazil, the number of hospitalizations and adverse outcomes, including death caused by COVID-19, in women during the pregnancy-puerperal cycle was high in the first pandemic year. Doubts regarding vaccines' efficacy and safety for the mother and fetus delayed vaccination. This study evaluated the generation of IgG titers and neutralizing antibodies to the BNT162b2 mRNA vaccine in 209 healthy pregnant women. For this, were used the QuantiVac ELISA (IgG) and SARS-CoV-2 NeutraLISA kits (EUROIMMUN, Lübeck, SH) following the manufacturer's recommendations. One dose vaccine produced anti-SARS-CoV-2 IgG in 85% (81/95), and two produced in 95% (76/80) women. Among unvaccinated women, four of 34 (12%) showed protection. The first dose of the BNT162b2 vaccine protected 69% of the women with neutralizing antibodies (median of %IH = 97). In the second dose, protection occurred in 94% of the pregnant women (median of IH% = 97). This study showed no differences in IgG antibody titers between one- and two-dose of the BNT162b2 mRNA vaccine groups, boosting with the second dose increased the number of women who produced specific IgG and neutralizing antibodies, raising by 114-folds the chance of producing the SARS-CoV-2 neutralizing antibodies compared to the unvaccinated pregnant woman, which may contribute to reduce the chance of severe COVID-19.

## 1. Introduction

Women have physiological changes in the immune and cardiorespiratory systems during pregnancy, which increases the risk of developing severe coronavirus disease (COVID-19) and eventual death. The unknown teratogenic effect of COVID-19 vaccines did not allow pregnant women to participate in the initial clinical trials. Nevertheless, after reports on COVID-19 deaths in pregnancy, the vaccine began to be administered in this vulnerable population before the scientific confirmation of its efficacy (1, 2).

In Brazil, the number of hospitalizations and adverse outcomes, including death caused by COVID-19, in women during the pregnancy-puerperal cycle were high in the first pandemic year (3, 4). In January 2021, the Brazilian Public Ministry of Labor and the Ministry of Health released a technical note recommending home office during pregnancy. However, despite the Brazilian National Health Surveillance Agency (ANVISA—Agência Nacional de Vigilância Sanitária) approval of the Sinovac/Coronavac™ and the Covishield/Oxford™ vaccines for emergency use, pregnant women were not a priority group for vaccination due to a scientific lack of vaccine's safety and efficacy in this population (5, 6). In June 2021, the National Immunization Program included pregnant and postpartum women as a priority group for vaccination. The ANVISA restricted the use of adenovirus vector based-vaccines Oxford/Covishield (Fiocruz/AstraZeneca™) and Janssen COVID-19 Vaccine (Janssen-Cilag™) because of the emergence of cases of Thrombosis with Thrombocytopenia Syndrome (TTS) (7). Given this, pregnant women were almost exclusively vaccinated with the Comirnaty vaccine (Pfizer/Wyeth™), the BNT162b2 mRNA-based vaccine, in which first studies were shown the production of neutralizing antibodies against SARS-CoV-2 and the absence of reproductive toxicity in an animal model (7–10).

Previous studies have shown that pregnant women produced IgG antibodies 5 days after the first dose, and the transplacental passive immunization occurred in 44% of neonates from the 16th day after the first dose of the mother's vaccine and in 99% of neonates of mothers who received two doses (1). Although there are some studies in the literature about this issue, mainly in USA and Israel, the immunogenicity after SARS-CoV-2 vaccination in Brazilian pregnant remains unknown. In this study, we evaluated the total IgG and the neutralizing IgG antibody levels in Brazilian pregnant women unvaccinated and vaccinated with one or two doses of the BNT162b2—Comirnaty mRNA vaccine (Pfizer/Wyeth™).

## 2. Materials and methods

### 2.1. Ethical issue and study population

The Instituto de Medicina Integral Prof. Fernando Figueira (IMIP) Ethics Committee approved this study (CAAE: 32359320.3.3001.5201). Pregnant women were invited to participate in this study during routine prenatal care consultations at IMIP Hospital between August and October 2021. All participants signed the Informed Consent Form after receiving detailed information about the study. The study population comprised 209 asymptomatic pregnant women aged between 18 and 42 years old, with an average age of 28. Previous SARS-CoV-2 infection was investigated following clinical criteria defined by the Brazilian Ministry of Health. The criteria associated with the circulating variant included fever, agnosia, anosmia, asthenia, flu syndrome, and a recent close contact with a laboratory confirmed case (Table 1).

**Table 1:** History of active and passive immunity against SARS-CoV-2 in pregnant women.

**History of COVID-19**	***N* = 209**	**100%**
No	143	68.4
Yes	66	31.6
**Infection period**	*N* = 66	100%
Before pregnancy	54	81.8
During pregnancy	12	18.2
**Infection confirmation**	*N* = 66	100%
Laboratory confirmed	31	47.0
Clinically suspected	35	53.0
**COVID-19 vaccination**	*N* = 209	100%
Pfizer 1 dose	95	45.4
Pfizer 2 doses	80	38.3
No vaccines	34	16.3

### 2.2. ELISA assays

Serological tests used plasma of patients recovered from the peripheral blood collected in an anticoagulant tube by centrifugation at 340 × g for 30 min. We used the anti-SARS-CoV-2 QuantiVac ELISA (IgG) kit (EUROIMMUN, Lübeck, SH) to quantify IgG class antibodies that bind the S1 subunit of the SARS-CoV-2 spike protein, following the manufacturer's instructions. Results were expressed in relative units per milliliter (RU/ml). The test was negative for values < 8 RU/ml and positive for values ≥11 RU/ml. Tests with intermediate values were considered borderline and inconclusive.

For the semi-quantitative assay of the IgG-neutralizing antibodies, we used the SARS-CoV-2 NeutraLISA kits (EUROIMMUN, Lübeck, SH). This kit detects antibodies capable of neutralizing the S1 subunit where the receptor binding domain (RBD) of the SARS-CoV-2 spike protein is located. Due to the neutralization kit's limited supply for evaluating all patients, we analyzed all 34 unvaccinated women and randomly selected 74 (77.9%) patients vaccinated with one and 72 (90%) with two doses. The results were in percent inhibition (%IH), and following the manufacturer's instructions, samples with %IH < 20 were negative, with %IH ≥ 35 were positive, and values in the middle range were borderline and inconclusive. The Multiskan FC Microplate Photometer (Thermo Scientific, Waltham, MA) was used to take the absorbance readings.

### 2.3. Statistical analysis

Descriptive data used absolute values and percentages; these data were previously evaluated for parametric or non-parametric approaches. In the analysis of quantitative variables, the Bartlett test was used to test the assumption of homogeneity of the variables involved in the study.

As the population proved to be heterogeneous, the Kruskal-Wallis test was used, followed by the Fisher's *post-hoc* test, which uses Fisher's least significant difference (LSD) criterion. In this way, the existence of differences in the medians for the IgG and %IH values was evaluated in relation to those without the vaccine, one dose, and two doses. Simple linear regression models were performed to assess IgG and %IH in relation to time with one and two doses of vaccine.

In the comparative analysis between the qualitative variables, the chi-square test was used and, Fisher Exact test when necessary. All conclusions were taken at the 5% significance level.

The statistical analyses were performed using the software of the R Core Team Version 4.2.1. (2022). A language and environment for statistical computing provided by the R Foundation for Statistical Computing, Vienna, Austria. URL https://www.R-project.org/.

## 3. Results

### 3.1. Anti-SARS-CoV-2 IgG titers in pregnant women after vaccination

**Figure 1:**
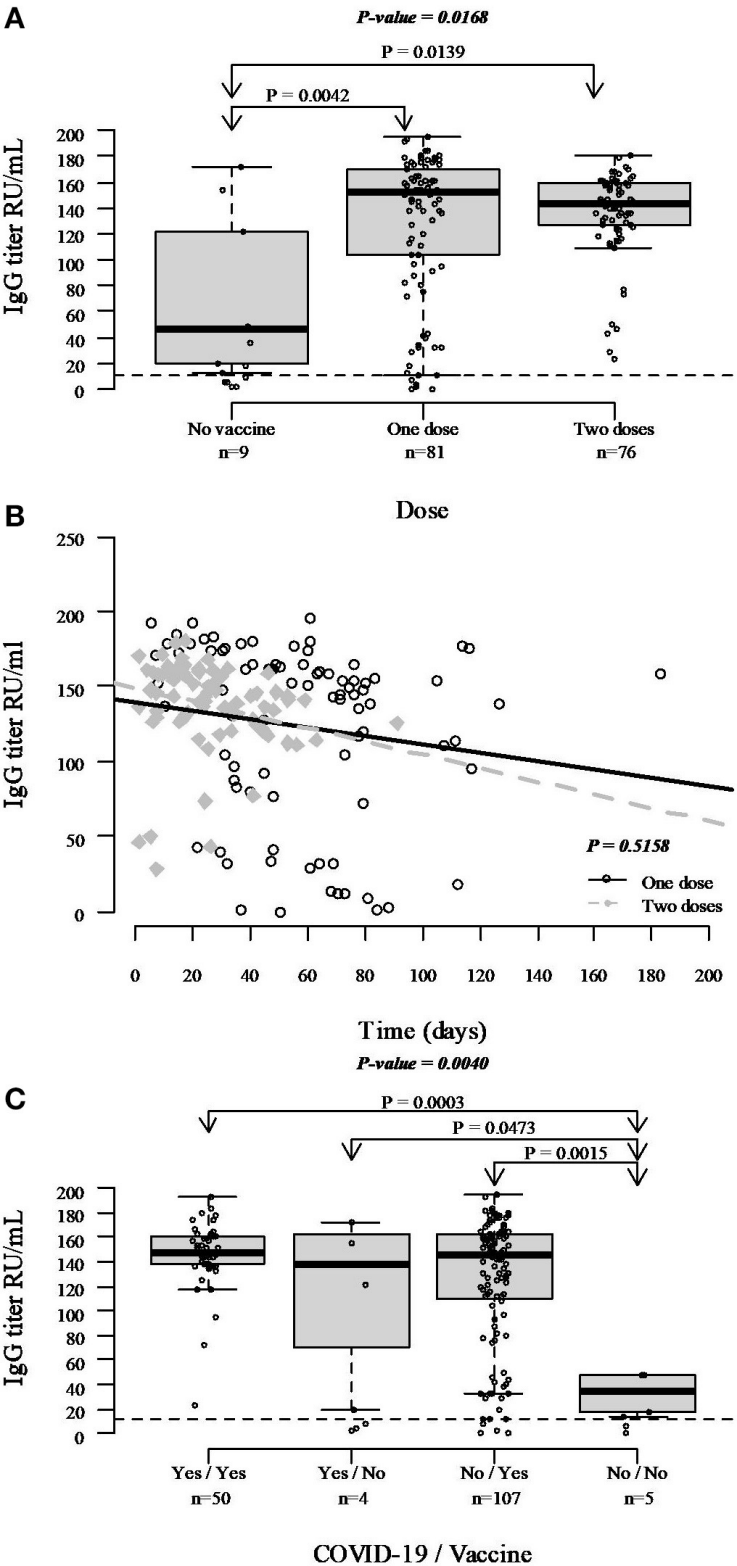
IgG levels against SARS-CoV-2 in unvaccinated pregnant women and vaccinated with one or two doses of mRNA vaccine. **(A)** Comparative analysis of the IgG title between positive samples (considering only IgG ≥11 RU/ml) from unvaccinated (9/34) and vaccinated with one (81/95) or two doses (76/80) pregnant women. The Bartlett's test analyzed the homogeneity of variances, and the Kruskal-Wallis test performed the statistics. **(B)** Comparative analysis of IgG antibody levels in patients vaccinated with one and two doses over time (considering only IgG ≥11 RU/ml) was performed using simple linear regression. **(C)** IgG antibody titers considering only IgG ≥11 RU/ml, history of SARS-CoV-2 infection, presence, and absence of vaccination. The Bartlett's test analyzed the homogeneity of variances, and the Kruskal-Wallis test performed the statistics.

Differences between the anti-SARS-CoV-2 IgG antibody levels generated by the natural infection and one or two vaccine doses were investigated (Figure 1A). Pregnant women without vaccines (with a history of virus infection) had lower production of specific IgG compared to pregnant women vaccinated with one dose (*P* = 0.0042) and two doses (*P* = 0.0139). However, the IgG levels generated by pregnant women who received one or two doses of the vaccine did not show a significant difference (*P* = 0.3722). The production of specific antibodies was observed in 85% (81/95) of pregnant women vaccinated with one dose and 95% (76/80) with two doses. As this is a cross-sectional study, a linear regression test was performed to evaluate IgG titers over time, and it was found that over a period of 180 days, there was no difference between the IgG titers generated by one or two doses (*P* = 0.5158), however, in the population with two doses, there is a greater tendency to decrease these titers over time (*P* = 0.0009) (Figure 1B).

When evaluating the impact of previous SARS-CoV-2 infections on the generation of IgG antibodies in vaccinated and unvaccinated people, we observed that some women who referred no previous infection and no vaccination presented low titer of IgG, suggesting previous mild or asymptomatic COVID-19 or cross-reaction due to infection by another circulating coronavirus; who were primed by infection and busted by vaccination presented the highest and homogeneous IgG levels; and natural infection induced more variable IgG levels (Figure 1C).

### 3.2. SARS-CoV-2 neutralizing antibodies after one or two vaccine doses

**Figure 2:**
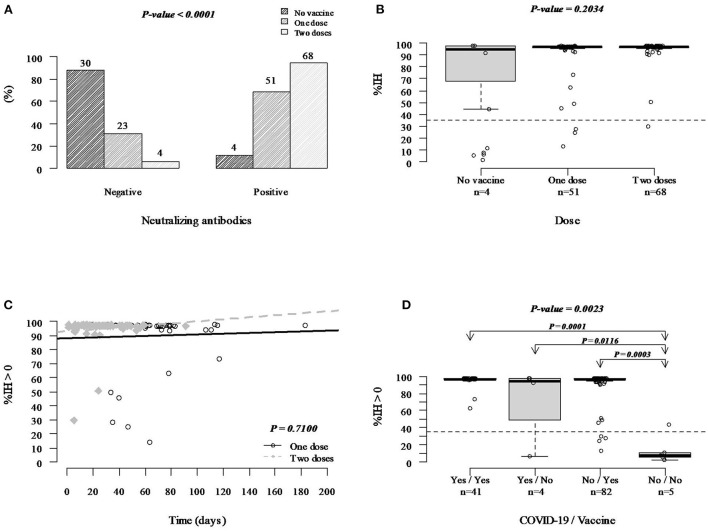
Protection against COVID-19 induced by the BNT162b2 mRNA-based vaccine according to the anti-SARS-CoV-2 neutralizing antibodies. **(A)** Protection and risk analysis among unvaccinated women and women vaccinated with one and two doses. **(B)** Comparative analysis of the neutralizing antibodies levels between positive samples (considering only %IH ≥ 35) from unvaccinated (4/34) and vaccinated with one (51/95) or two doses (68/80) pregnant women. The Bartlett's test analyzed the homogeneity of variances, and the Kruskal-Wallis test performed the statistics. **(C)** Comparative analysis of neutralizing antibody levels in patients vaccinated with one and two doses over time (considering only %IH ≥ 35) was performed using simple linear regression. **(D)** Neutralizing antibody titers considering only %IH > 0, history of SARS-CoV-2 infection, presence and absence of vaccination. The Bartlett's test analyzed the homogeneity of variances, and the Kruskal-Wallis test performed the statistics.

The anti-SARS-CoV-2 neutralizing antibodies assay investigated the possible protection against COVID-19 from pregnant women's vaccination. The first dose of the BNT162b2 vaccine protected 69% of the women (*n* = 51/74, median of %IH = 97), while after the second dose, protection occurred in 94% of the pregnant women (*n* = 68/72, median of %IH = 97). Among unvaccinated women, sole 12% showed protection (*n* = 4/34, median of %IH = 95), suggesting immunological memory of the previous contact with the virus (Figures 2A, B). It was observed that there is no difference in the %IH of neutralizing antibodies between the groups of unvaccinated and vaccinated with one and two doses (*P* = 0.2034) (Figure 2B). It was also verified that there is no difference between the %IH between the groups with one and two doses over 180 days (*P* = 0.7100), however, when linear regression was performed, it was seen that there is a maintenance in the %IH generated by neutralizing antibodies over time in the groups with one (*P* = 0.742) and two doses (*P* = 0.291) (Figure 2C). When the impact of previous SARS-CoV-2 infection on the generation of neutralizing antibodies in the groups of unvaccinated and vaccinated women was evaluated, it was seen that who referred no previous infection and no vaccination produced very low neutralizing antibody titers, with only one sample being positive according to the test cut-off, and who had SARS-CoV-2 natural infection or received COVID-19 vaccines, associated or not, presented significant higher levels (Figure 2D).

**Figure 3:**
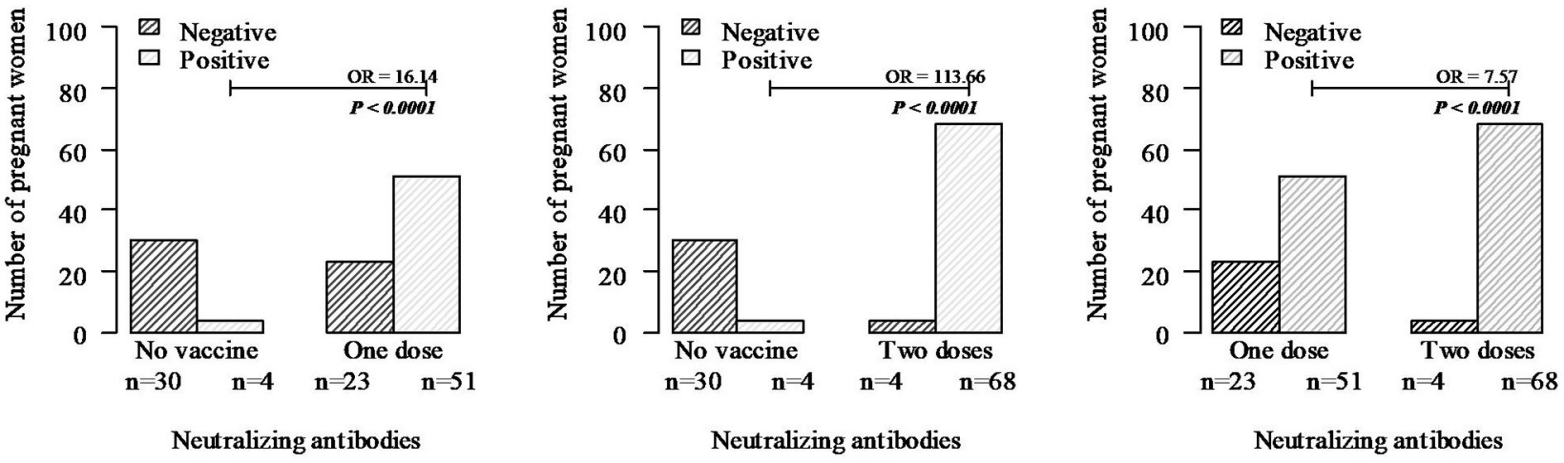
Protection conferred by neutralizing antibodies in unvaccinated pregnant women and vaccinated with one and two doses of the BNT162b2 mRNA-based vaccine. Neutralizing antibodies were quantified from all 34 unvaccinated women and 74 women vaccinated with one dose and 72 with two doses selected randomly. Statistics were performed with chi-square, and when necessary, Fisher's exact test.

Women who received one and two doses of BNT162b2 mRNA-based COVID-19 vaccine had, respectively, a 16-fold and 114-fold chance to produce neutralizing antibodies above the method cut-off compared to unvaccinated pregnant women, while pregnant women vaccinated with two doses had a 7-fold chance to produce neutralizing antibodies above the method cut-off when compared with the group with one dose (Figure 3).

## 4. Discussion

This study showed no differences in IgG antibody titers between one- and two-dose of the BNT162b2 mRNA vaccine groups; boosting with the second dose increased the number of women who produced specific IgG and neutralizing antibodies, contributing to reducing the chance of severe COVID-19 by 114-fold compared to unvaccinated pregnant women. According to epidemiological bulletins published by the Pernambuco State Health Department during this study, from August to October of 2021, the SARS-CoV-2 variant Gamma P.1 corresponded to 74% of cases in August, while between September and October, 87% of the population was infected by the Delta variant (11). The Strategic Advisory Group of Experts in Immunization (SAGE) of the WHO reported that the BNT162b2 mRNA vaccine is efficacy against all SARS-CoV-2 circulating variants (12). These results corroborate the findings of Pratama et al. (2) and Andreano and Rappuoli (13) who defend the complete vaccination schedule for protection.

Beharier et al. (14) demonstrated in a group of 86 Israeli pregnant women with no history of SARS-CoV-2 infection that the BNT162b2 mRNA-based vaccine [Comirnaty vaccine (Pfizer/Wyeth™)] increased IgG titers and adequately transferred them across the placenta, inducing an anti-SARS-CoV-2 protective effect in the neonate's bloodstream as early as two weeks after the first dose. In addition, the protective immune response induced by mRNA vaccines and transferred as IgG and IgA antibodies by breastfeeding were more efficient than the response to natural infection in pregnant women (15). We also showed that although 26.5% (9 of 34) unvaccinated pregnant women had IgG against COVID-19, suggesting previous exposure, less than half had neutralizing antibodies (4 of 9). For unknown reasons, not all those infected with SARS-CoV-2 develop neutralizing antibodies, and when they do, they are not always sufficient to protect the patient against the virus (16, 17).

A limitation of our study was the impossibility of longitudinally monitoring the levels of neutralizing antibody. Although we do not know for how long the neutralizing antibodies levels remain elevated in pregnant women, our data suggested that the BNT162b2 mRNA-based vaccine induced high levels of neutralizing antibodies that remains stable longer than IgG titers. A cohort study of 4,868 healthcare workers in Israel reported a significant reduction in the IgG and neutralizing antibodies titers after six months of the boosting with the BNT162b2 vaccine, especially in the male and elderly population (18). Another study involving 6,056,673 English people also reported a decline in the effectiveness of the BNT162b2 (Comirnaty vaccine, Pfizer/Wyeth™) and ChAdOx1-S (Oxford/Covishield, Fiocruz/AstraZeneca™) vaccines in hospitalization and death after about 20 weeks, mainly in the elderly (>65 years) and some clinical risk groups (19).

We did not evaluate the safety of the BNT162b2 vaccine for the mother and neonate, but given the results reported in this study and the recognized risks for the woman and the fetus of COVID-19 during pregnancy, pregnant women must continue to be encouraged to receive the COVID-19 vaccine as it is a way to protect their fetuses during pregnancy and probably also during the breastfeeding period (20–22).

## Data availability statement

The raw data supporting the conclusions of this article will be made available by the authors, without undue reservation.

## Ethics statement

The studies involving human participants were reviewed and approved by the Instituto de Medicina Integral Prof. Fernando Figueira (IMIP) Ethics Committee approved this study (CAAE: 32359320.3.3001.5201). The patients/participants provided their written informed consent to participate in this study.

## Author contributions

MS and NL-S conceived, designed the study, did the formal analysis, and wrote the paper. MS, NS, JM, and GD conducted the experimental work. MS, MM, FF, CB, and LS performed the clinical interview and patients followed up. AF, AS, and NL-S applied for financial support and managed the project. All authors contributed to the article and approved the submitted version.
